# Targeting DLBCL by mutation-specific disruption of cancer-driving oncogenes

**DOI:** 10.3389/fgeed.2024.1427322

**Published:** 2024-10-14

**Authors:** Najmeh Heshmatpour, S. Maryam Kazemi, Niklas D. Schmidt, Sarita R. Patnaik, Patrick Korus, Bodo G. C. Wilkens, Arturo Macarrón Palacios

**Affiliations:** GenCC GmbH & Co. KG, Heidelberg, Germany

**Keywords:** CRiSPR/Cas, cancer, DLBCL, gene knockout, precision medicine, mutation-specific gRNA, crRNA

## Abstract

Diffuse large B cell lymphomas (DLBCL) are highly aggressive tumors. Their genetic complexity and heterogeneity have hampered the development of novel approaches for precision medicine. Our study aimed to develop a personalized therapy for DLBCL by utilizing the CRISPR/Cas system to induce knockouts (KO) of driver genes, thereby causing cancer cell death while minimizing side effects. We focused on OCI-LY3 cells, modeling DLBCL, and compared them with BJAB cells as controls. Analysis of whole exome sequencing revealed significant mutations in genes like *PAX5*, *CD79B*, and *MYC* in OCI-LY3 cells. CRISPR/Cas9-mediated KO of these genes resulted in reduced cancer cell viability. Subsequent single and dual gRNA targeting of *PAX5* mutations inhibited proliferation specifically in OCI-LY3 cells. Moreover, dual gRNA targeting of *PAX5* and *MYC* induced chromosomal rearrangements, reducing cell proliferation substantially. However, targeting single intronic mutations did not affect cell viability, highlighting the importance of disrupting protein function. Targeting multiple mutations simultaneously addresses intra-tumoral heterogeneity, and the transient delivery of CRISPR/Cas9 allows for permanent gene disruption. While challenges such as incomplete editing efficiency and delivery limitations exist, further optimization may enhance therapeutic efficacy. Overall, our findings demonstrate the efficacy of CRISPR/Cas9 in targeting oncogenic mutations, opening avenues for precision medicine in DLBCL treatment.

## Introduction

According to WHO reports, cancer is a leading cause of death on a global scale with nearly one in every six deaths (Ferlay et al., 2021). This fatal illness is brought on by a progressive formation of mutations and epigenetic modifications in the cellular genome, which results in uncontrolled growth, resistance to tumor suppressors and cell death indications, and an increase in genetic variation throughout the tumorigenesis cycle (Balon et al., 2022). The cancer of the cells that make up the lymphatic system is known as lymphoma and includes many manifestations, one example being diffuse large B-cell lymphoma (DLBCL) (Taguchi et al., 2021), the most common aggressive non-Hodgkin´s lymphoma (NHL). Despite the potent effect of standard treatment regimens, around 35% of patients still show drug resistance or recurrence after remission, inevitably leading to poor prognosis and low survival rates in these patients (Zhang et al., 2023). Furthermore, both treatment resistance and disease prognosis are strongly influenced by the tumor microenvironment, as several components in the environment can promote not only immune escape and proliferation but also tumor cell migration, leading to residual disease and relapse (Fornecker et al., 2019; Solimando et al., 2020).

The cancer cells in DLBCL are morphologically and molecularly heterogeneous, as they accumulate genetic mutations that enable them to grow and survive (Takahara et al., 2023). Two main DLBCL subtypes are recognized, the germinal center B-cell-like (GCB) and the activated B-cell-like (ABC) subtype. The cells of the former subtype have a gene expression profile related to a germinal center (GC) cell of origin and are characterized by enrichment of *IGH::BCL2* fusion and mutations of genes crucial for GC development, such as *EZH2*, *GNA13*, *MEF2B*, *KMT2D*, *TNFRSF14*, *B2M,* and *CREBBP*. In contrast, the ABC subtype displays rather a germinal center-exit or early plasmablastic phenotype, as it derives from cells of GC exit. These cells are typically dependent on B-cell receptor (BCR) and NFκB signaling and are enriched for BCR pathway mutations such as in in *MYD88*, *CD79B,* and *PIM1* (Katti et al., 2022). Only through understanding the gene and mutation profile for this disease will it be possible to develop drugs targeted explicitly against the malignant cells (Taguchi et al., 2021).

The emergence of CRISPR/Cas9 technology is a significant advancement in genetic engineering, offering a highly effective method for gene manipulation. CRISPR, short for Clustered Regularly Interspaced Short Palindromic Repeats, serves as the genetic component, while Cas9, a bacterial enzyme, aids in the editing process (Doudna and Charpentier, 2014). The CRISPR/Cas9 system primarily involves a single-guide RNA (sgRNA) and an RNA-guided Cas9 endonuclease. The Cas9 protein comprises two distinct nuclease domains, HNH and RuvC, which cleave a single strand of the targeted double-stranded DNA (Chira et al., 2022). The trans-activating CRISPR RNA (tracrRNA) and the CRISPR RNA (crRNA) combine to form single-guide RNAs. These, along with the Cas9 nuclease, generate the Cas9 ribonucleoprotein (RNP), capable of binding and cleaving the targeted DNA. Repair of the double-stranded breaks (DSBs) induced by genome editing procedures occurs through either the error-prone non-homologous end joining (NHEJ) pathway or the homology-directed repair (HDR) pathway (Chira et al., 2022). NHEJ, operating in approximately ninety percent of cell sequences, does not require a close homologous donor and is generally more productive than HDR. It may result in random insertions and deletions (InDels) at cleavage sites, leading to premature stop codons or frameshift alterations within the targeted genes' open reading frames (ORFs), rendering them inactive (Xu and Li, 2020). Conversely, HDR employs a homologous DNA repair template to precisely induce genomic alterations at the target site (Honeywell et al., 2023).

The CRISPR/Cas genome-editing technology holds multitudinous potential applications across several fields, including medicine, agriculture, and biotechnology. These include gene editing, animal modeling, drug discovery, library generation, RNA targeting, cancer dependency mapping, and immune cell engineering, among many others (Tsimberidou et al., 2012; Yang et al., 2021; Palacios et al., 2024; Mengstie and Wondimu, 2021). During the last years, researchers have focused on correcting or disabling genomic aberrations leading to different diseases such as Duchenne muscular dystrophy (DMD) (Long et al., 1979; Russell et al., 2022), transthyretin amyloidosis (Kotit, 2023), beta-thalassemia, sickle cell disease (Frangoul et al., 2021), Leber congenital amaurosis and other inherited retinal dystrophies (Russell et al., 2022).

In the cancer field, CRISPR/Cas is not only crucially instrumental as a diagnostic tool (Kim et al., 2021; Liu et al., 2023) and for the development of adoptive *ex vivo* therapies (Yang et al., 2021; Liu et al., 2023; Johnson et al., 2018; Zhang et al., 2018; Wang and Song, 2017), but also for the direct targeting of malignant cells. Recent advancements emphasize the importance of targeting common mutations, such as those in the epidermal growth factor receptor (EGFR), particularly the L858R variant within the tyrosine kinase domain implicated in lung cancer. The innovative CRISPR/Cas9 methodology enables precise manipulation of *EGFR* mutations, resulting in efficient tumor regression (Hille et al., 2018; Cheung et al., 2018). Further evidence on targeting frequent mutations is provided by studies targeting missense mutations in codon-12 of the *KRAS* oncogene (Lee et al., 2018; Gao et al., 2020). Other therapeutic strategies are the targeting of multiple InDels, resulting in the induction of many DSBs and therefore severe DNA damage in a cancer cell-specific fashion (Kwon et al., 2022a), and the disruption of viral oncogenes (Jubair et al., 2019) or fusion oncogenes (FOs) (Martinez-Lage et al., 2020).

For DLBCL, in particular, recent research suggests a broader array of genetic alterations contributing to cancer development and progression. These mutations, found in genes associated with various cellular pathways, collectively shape the disease phenotype and therapeutic responses (Reddy et al., 2017). For instance, *MYC* dysregulation is implicated in aberrant cell cycle progression and therapy resistance (Kalkat et al., 2017). Similarly, *PAX5* mutations may disrupt B-cell differentiation and confer resistance to conventional therapies (Schebesta et al., 2007). Additionally, mutations in *CD79B* have been linked to constitutive B-cell receptor signaling and treatment resistance (Conacci-Sorrell et al., 2014). While these genes are recognized contributors to DLBCL pathogenesis, emerging evidence suggests that other genetic aberrations, including alterations in epigenetic regulators and immune-related genes, also play pivotal roles (Reddy et al., 2017). Understanding the broader genomic landscape of DLBCL is crucial for developing targeted therapies and improving patient outcomes.

This study aims to explore the efficacy of guide RNAs designed against mutations associated with diffuse large B-cell lymphoma in inducing cancer cell death. Employing an *in vitro* experimental approach, we investigate the impact of the CRISPR/Cas9 technology on cell lines harboring DLBCL-associated mutations, known for their aggressive nature. Specifically, we target mutations within two key oncogenic molecules, *PAX5* and *MYC*, aiming to elucidate their role in triggering cancer cell death using the CRISPR-Cas9 system.

## Methods

### Whole exome sequencing analysis of OCI-LY3 and BJAB cell lines

Whole exome sequencing (WES) data for OCI-LY3 and BJAB cell lines were obtained from the DSMZ (www.dsmz.de) and are publicly available in the European Nucleotide Archive (ENA) under the accession number PRJEB30297. WES reads of DLBCL samples were mapped against the *Homo sapiens* reference genome GRCh38.p13 from the Genome Reference Consortium [GCA_000001405.28 & GCF_000001405.39]. Germline mutations were thereby sorted out. The alignment was conducted using the BWA MEM tool in the Galaxy platform (https://usegalaxy.org/), resulting in Binary Alignment Map (BAM) files. The BAM files were utilized for the identification of structural variants (SVs) and single-nucleotide variants (SNVs) using the FreeBayes tool in Galaxy. This analysis produced Variant Call Format (VCF) files, allowing for comprehensive variant assessment.

### Cell lines and culturing conditions

OCI-LY3 (ACC 761) and BJAB (ACC 757) cell lines, were sourced from DSMZ. The provided instructions were followed for culturing these cells in RPMI 1640 Medium (ATCC modification) (A1049101; Thermo Fisher Scientific) with 20% heat-inactivated fetal bovine serum (FBS) (A3160502; Thermo Fisher Scientific) and 1% penicillin-streptomycin solution (10378016; Thermo Fisher Scientific). The cell cultures were maintained in an incubator at 37°C with 5% CO_2_ to facilitate optimal growth.

### Guide RNA design

For designing gRNAs targeting mutations, the SNP-CRISPT tool (https://www.flyrnai.org/tools/snp_crispr/) was employed. This tool allows the upload of multiple single-nucleotide polymorphisms (SNPs) to design gRNAs targeting single or multiple nearby mutations concurrently. It generates all possible gRNAs for a variant based on the positioning of PAM. Variants that introduce new PAM sequences were manually designed. Additionally, the Custom Alt-R^®^ CRISPR-Cas9 guide RNA tool from IDT was utilized to assess the gRNAs on-target and off-target scores.

### Nucleofections

The ribonucleoprotein (RNP) complex was assembled following the manufacturer’s guidelines. Briefly, each Alt-R crRNA (IDT) and Alt-tracrRNA-ATTO550 (1072533, IDT) was reconstituted to a concentration of 100 µM using Nuclease-Free Duplex Buffer (11-05-01-14, IDT). The crRNA and tracrRNA oligos were combined in equimolar concentrations, resulting in a final duplex concentration of 44 μM. The oligos were annealed by heating at 95°C for 5 min and slowly cooled to room temperature. The crRNA–tracrRNA duplex (sgRNA) and Alt-R^®^ S.p. HiFi Cas9 Nuclease 3NLS (1078727, IDT) were precomplexed by gentle mixing and incubated at room temperature for 10–20 min sgRNAs were complexed with Cas9 at a molar ratio ranging from 1:1 to 3:1 (sgRNA:Cas9) to form RNPs, freshly prepared for each experiment. To enhance transfection efficiency, Alt-R electroporation enhancer (IDT) was added to the mix at a final concentration of 1.75 μM. Approximately 5 × 10^5^ cells were resuspended in Neon electroporation buffer R and electroporated using the 10 μL Neon transfection system kit (Thermo Fisher Scientific), employing two pulses at 1400 V with a width of 20 ms. Transfected cells were then incubated for 48–72 h in pre-warmed RPMI-1640 medium supplemented with 20% FBS (A3160502; Thermo Fisher).

### Determination of editing efficiency and gene deletion/rearrangement

Genomic DNA extraction was carried out by lysing cells using QuickExtract™ DNA Extraction Solution (Lucigen; Biozym QE09050). PCR primers were designed for each target to amplify the flanking region of the targeted genomic DNA, producing a PCR amplicon of <900 bp. GoTaq^®^ G2 Hot Start Taq Polymerase (M5122; Promega) was utilized for PCR to screen for InDels/mutations in the region of interest. The resulting PCR amplicon underwent purification using either PureLink^®^ PCR Purification Kit (Invitrogen; K3100-02) or NucleoSpin Gel and PCR Clean-up kit (740609.250; Macherey-Nagel).

Sanger sequencing was conducted by a commercial vendor (Eurofins; Heidelberg) using one of the two primers used for amplification. Sequence traces obtained from Sanger sequencing were analyzed with the Interference of CIRPSR Edits (ICE) tool (https://ice.synthego.com/), which calculates overall editing efficiency and identifies the profiles of various CRISPR edits. ICE default parameters provided InDel patterns and their relative ratios, enabling alignment of the non-targeting control (NTC) trace sequence with a gene-specific sgRNA-edited trace sequence. This alignment allowed visualization of the InDel patterns in each polyclonal population.

PCR analysis was employed in experiments using two different RNPs targeting unique regions of the same or different genes, resulting in deletions and cancer-specific chromosomal rearrangements. The confirmation of deletions or rearrangements was achieved by designing primers spanning the region to be deleted. In cells edited with dual gRNAs, the PCR band was only amplified if both crRNAs were successfully edited. Without dual editing and subsequent rearrangement, the primers failed to generate any PCR product.

### Cell viability, proliferation and cell death assessment

For cell viability assessment, immediately following nucleofection, cells were seeded in a 96-well plate with a total volume of 100 μL medium, including RealTime-Glo MT cell viability reagents (RealTime-Glo MT Cell Viability Assay; Promega). Cells were then incubated in a 37°C, 5% CO_2_-humidified incubator (Thermo Fisher Scientific). After 1 hour, luminescence was measured using a SpectraMax^®^ iD3 multi-mode microplate reader (Molecular Devices) set at 37°C. Additional readings were taken from the same plate at 72 h post-nucleofection. The luminescence at 72 h was normalized to the 1-h reading to account for any variations resulting from pipetting.

For cell death, cells were immediately seeded in a 96-well plate with a total volume of 100 μL medium following nucleofection, including CellTox™ Green Dye 8 (CellTox™ Green Cytotoxicity Assay; Promega). Cells were then incubated in a 37°C, 5% CO_2_-humidified incubator (Thermo Fisher Scientific). After 30 minutes, fluorescence was measured using a SpectraMax^®^ iD3 multi-mode microplate reader (Molecular Devices) set at 37°C. Additional readings were taken from the same plate at 72 h post-nucleofection. The fluorescence at 72 h was normalized to the 30-min reading to account for any variations resulting from pipetting.

To measure cell proliferation, after 72 h, CellTiter^®^ 96 AQ_ueous_ reagent was added (CellTiter 96^®^ AQ_ueous_ One Solution Cell Proliferation Assay; Promega) to another 96-well plate seeded with the same cells after nucleofection. Luminescence was measured using a SpectraMax^®^ iD3 multi-mode microplate reader (Molecular Devices) set at 37 °C after 15 min, 30 min and 1 h of further incubation.

### Statistical analysis

Statistical analysis was conducted using GraphPad Prism software (GraphPad Software Inc., San Diego, CA, United States). Multiple group comparisons were carried out using ANOVA, followed by either Tukey’s, Dunnett’s, or Sidak’s multiple comparison test, depending on the specific data comparisons. Details regarding statistical tests and the number of repetitions is provided in the legends. Bar plots depict the mean ± standard error mean (SEM).

## Results

### Mutation profile analysis of OCI-LY3 cells for developing a targeted therapy development in DLBCL

Our study aims to develop a patient-tailored therapy with reduced side effects by utilizing the CRISPR/Cas system to induce knockouts (KO) of driver genes, consequently reducing cell viability. This personalized approach identifies mutations present in cancer cells but not in healthy cells. To this end, we selected OCI-LY3 as a model for their resemblance to cancer cell characteristics and compared them with BJAB cells, serving as a standard control.

First, we analyzed whole exome sequencing (WES) data of these cell lines to unravel their mutation profile. We identified around 362,000 mutations in 18,919 genes in OCI-LY3, including *PAX5*, *CD79B*, *CARD11*, *PIM1*, *mTOR*, and *BCL2,* genes frequently mutated in DLBCL patients (Supplementary Table S1). We also identified around 420,000 mutations in 20,040 genes in BJAB cells (Supplementary Table S2). Initially, we planned to test our approach with three crucial genes–*PAX5*, *MYC*, and *CD79B* –- known to play pivotal roles in cancer development and DLBCL growth.

This analysis unveiled a spectrum of mutations in OCI-LY3 cells, encompassing 65 mutations in *PAX5*, four mutations in *MYC*, and four mutations in *CD79B*. These mutations underwent further validation through Sanger sequencing (Supplementary Table S4). Notably, 45 mutations in *PAX5*, three mutations in *MYC*, and three mutations in *CD79B* were situated adjacent to or formed new protospacer adjacent motif (PAM) sequences for SpCas9.

For some mutations, multiple gRNAs could be designed. In this case, we prioritized those gRNAs where the target mutation was located within the first seven nucleotides, i.e., with the shortest distance to the PAM, or those gRNAs for which the mutation was positioned within the PAM sequence. This approach is supported by previous studies indicating that Cas9 can tolerate mismatches between the gRNA and the genome if they are distant from the PAM sequence, resulting in gene cleavage (Jinek et al., 1979). Consequently, guide RNAs were specifically designed to target these mutations (Supplementary Table S3).

To choose the final mutation-specific guide RNAs for the study, we considered several criteria such as the variant position, distance to PAM, or the formation of SNP-derived PAMs. Since we aimed to knock out essential genes and prevent gene translation, we prioritized exonic regions that would form the final mature RNA coding for a protein over intronic regions. Additionally, early exons (i.e., exons located in the first half of the gene) were also prioritized. Early splicing sites or splicing regulatory elements (intronic regions guiding exon splicing) were also given priority. Another strategy involved employing gRNA targeting mutations in early introns in combination with gRNA in late exons (i.e., exons located in the last half of the gene) to excise a large part of the target gene (Supplementary Figure S1).

### CRISPR/Cas9-mediated depletion of *MYC*, *PAX5*, and *CD79B* genes in DLBCL cells: implications for lymphoma therapy

To investigate the general feasibility of our approach, we first investigated the impact of functional driver gene knockout on cancer cell viability in the DLBCL cell line OCI-LY3 in a non-mutation-specific manner. To ensure the reliability and efficacy of the CRISPR/Cas9 system in our experimental setup, various optimization steps comprising nucleofection settings (voltage, pulse width, and pulse number), gRNA concentration, and incubation time after nucleofection were conducted (Supplementary Figure S2). The established protocol was used for all subsequent experiments.

**FIGURE 1:**
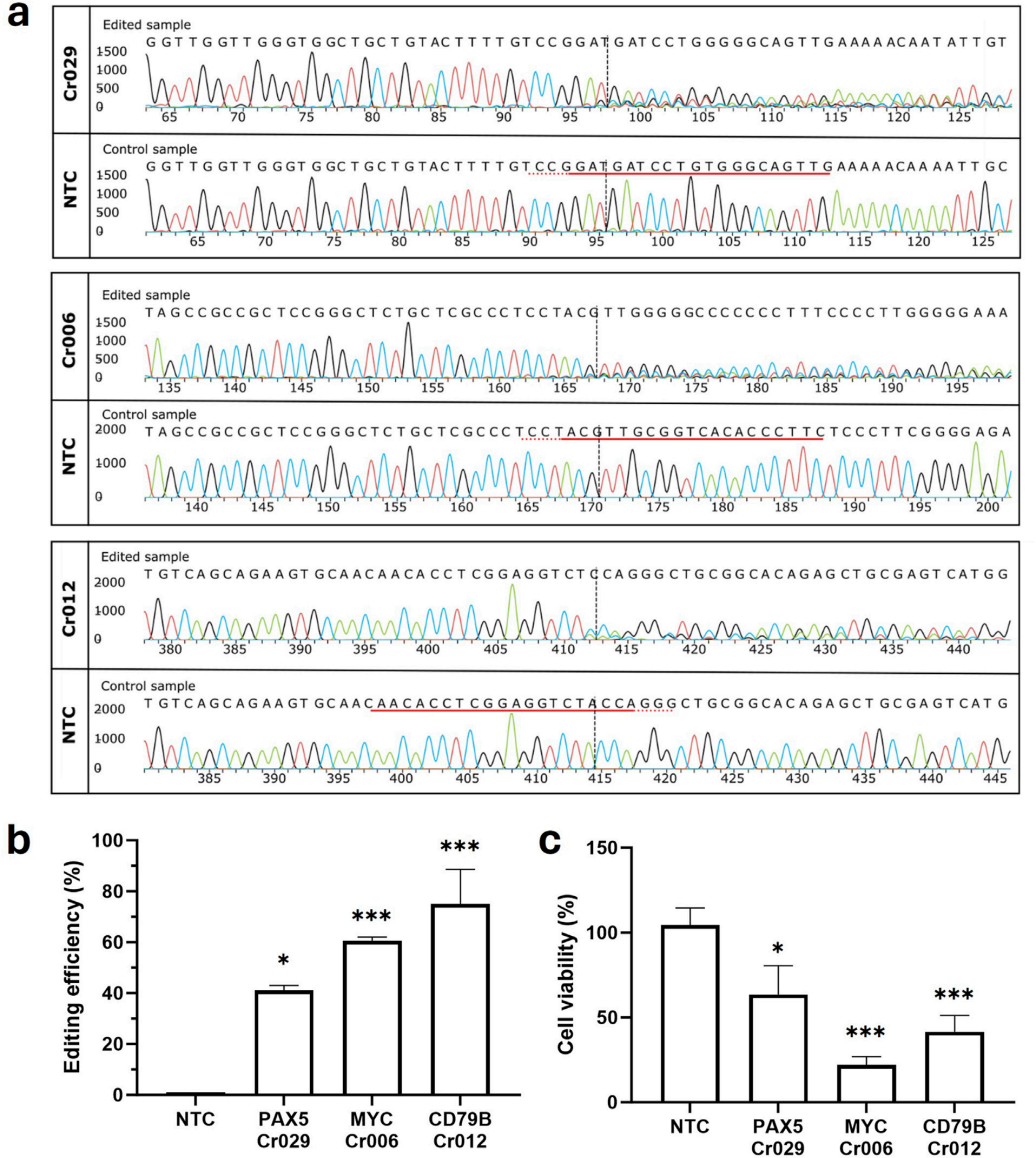
CRISPR/Cas9-mediated depletion of *MYC*, *PAX5*, and *CD79B* genes reduces cell viability in OCI-LY3 cells. Following electroporation of OCI-LY3 cells with crRNAs targeting non-mutated gene regions, genomic DNA was isolated after 72 h of treatment. Amplification of target sequence regions was performed using primers flanking the expected edited points, followed by Sanger sequencing. Editing efficiencies were calculated by comparing sequencing chromatograms of test samples with control cells treated with non-targeting crRNA (NTC) using the ICE web tool. The edited and control sanger traces around the guide RNA binding sites are illustrated. The horizontal black underlined region represents the guide sequence, along with the dotted gray underlined PAM site. The cut site is depicted by a vertical black dotted line **(A, B)**. Cell viability, determined as a percentage compared to the negative control, revealed significant reductions in viability for cells electroporated with Cr006, Cr029, and Cr012 **(C)**. Results are presented as mean ± SEM, with statistical significance indicated (*p < 0.05, ***p < 0.001).

We employed crRNAs to direct the CRISPR/Cas9 complex towards non-mutational regions within early exons of the genes *PAX5* (Cr029), *MYC* (Cr006) and *CD79B* (Cr012). The RNPs were introduced into OCI-LY3 cells via nucleofection. Subsequently, genomic DNA was isolated and screened for site-specific gene modifications by PCR followed by Sanger sequencing. Editing efficiency was quantified using Interference of CRISPR Edits (ICE) analysis. High mean editing levels ranging from around 40%–80% were observed for all three genes (Figures 1A, B). Moreover, CRISPR/Cas9-mediated editing resulted in significantly reduced viability of cancer cells compared to samples treated with a non-targeting control crRNA (NTC). Disruption of *CD79B* and *MYC* showed a notably higher impact on cancer cell survival, diminishing the cell viability up to 42% and 22%, respectively (Figure 1C). These findings suggest that depletion of these driver genes may represent a promising strategy for lymphoma treatment, potentially interfering with tumor growth and survival pathways not targeted by standard therapies. Moreover, these results serve as positive controls for future experiments reducing cell viability specifically in cancer cells using mutation-specific guide RNA in driver genes.

### CRISPR/Cas9-mediated targeting of *PAX5* mutation specifically reduces the number of viableOCI-LY3 cells

**FIGURE 2:**
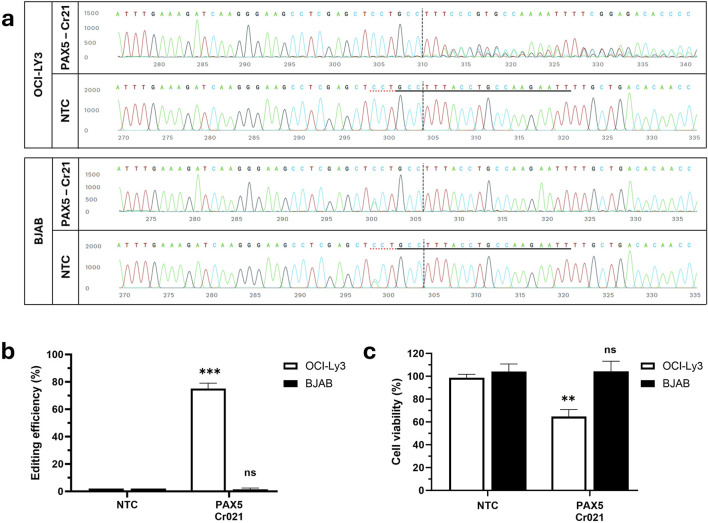
Knockout of the *PAX5* gene using OCI-LY3 specific-crRNA reduces cell viability. OCI-LY3 and BJAB lymphoma cells (2.5 × 10^5^ cells) were electroporated with Cr021, targeting the exon two-intron junction. After 72 h of treatment, cells were collected, and genomic DNA was isolated. The target sequence regions were amplified with primers flanking the expected edited point, followed by Sanger sequencing of the amplicon. The traces illustrate the edited and control (non-edited) Sanger traces around the gRNA binding site(s). The horizontal black underlined region represents the guide sequence, along with the dotted gray underlined PAM site. The cut site is marked by a vertical black dotted line **(A)**. Total editing efficiencies were then calculated by comparing the sequencing chromatogram of the test sample with control cells treated with non-targeting crRNA (NTC) using the ICE web tool **(B)**. Cell viability was determined as a percentage of viability from the negative control in OCI-LY3 and BJAB lymphoma cells electroporated with Cr021 **(C)**. Results are presented as mean ± SEM, with statistical significance indicated (**p < 0.01, ***p < 0.001).

Having confirmed the feasibility of mediating cancer cell death through gene disruption, we intended to induce cell death specifically in OCI-LY3 cells by employing a single mutation-specific crRNA. To disrupt the *PAX5* gene specifically in OCI-LY3 cells, we selected the *PAX5* variant A>C at position 37,020,625 (exon2-intron junction), which creates a new protospacer adjacent motif (PAM). This variant is homozygous in OCI-LY3 cells and heterozygous in BJAB cells. We designed a guide RNA, named Cr021, to target this mutation. We anticipated that the CRISPR/Cas9-gRNA complex would align with the variant-containing target regions in the *PAX5* gene, inducing a double-stranded DNA break. Subsequent repair attempts by the cell may lead to genetic frameshifts and the production of non-functional transcripts, ultimately resulting in the knockout of *PAX5*. OCI-LY3 and BJAB cells were nucleofected with ribonucleoprotein (RNP) complexes consisting of Cas9 protein and gRNA (Cr021). Interference of CRISPR edits (ICE) analysis revealed _∼_75% efficient editing of *PAX5* in OCI-LY3 cells (Figures 2A, B). Viability assays performed 72 h post-transfection showed a 35% reduction in viability. According to our expectations, no editing or impairment of cell viability was observed in BJAB cells (Figure 2C). This might be reasoned due to the presence of a wild-type gene copy in BJAB cells, which serves as a template upon editing and allows for gene correction. These findings demonstrate the efficacy of CRISPR/Cas9 in targeting specific mutations in early exons of cancer cells, leading to cancer-specific reduction of cell viability.

### CRISPR/Cas9-mediated targeting of *PAX5* mutations using dual gRNAs reduces the number of viable OCI-LY3 cells

Encouraged by the promising results observed with the mutation-specific crRNA Cr021, we next aimed to enhance the therapeutic efficacy of our approach by targeting multiple mutations within the same gene. While Cr021 addressed mutations in early exons, we purposed to tackle the challenge posed by mutations located in introns or late exons. Our objective was to induce large deletions, ultimately depleting the encoded protein. To this end, we employed two separate gRNAs, Cr021 and Cr022, targeting distinct regions of the *PAX5* gene. Cr022, targeting the last exon of the *PAX5* gene, specifically addressed the homozygous mutation G>T located at position 36,840,626 in OCI-LY3 cells but absent in BJAB cells. OCI-LY3 and BJAB cells were nucleofected with a combination of both gRNAs.

**FIGURE 3:**
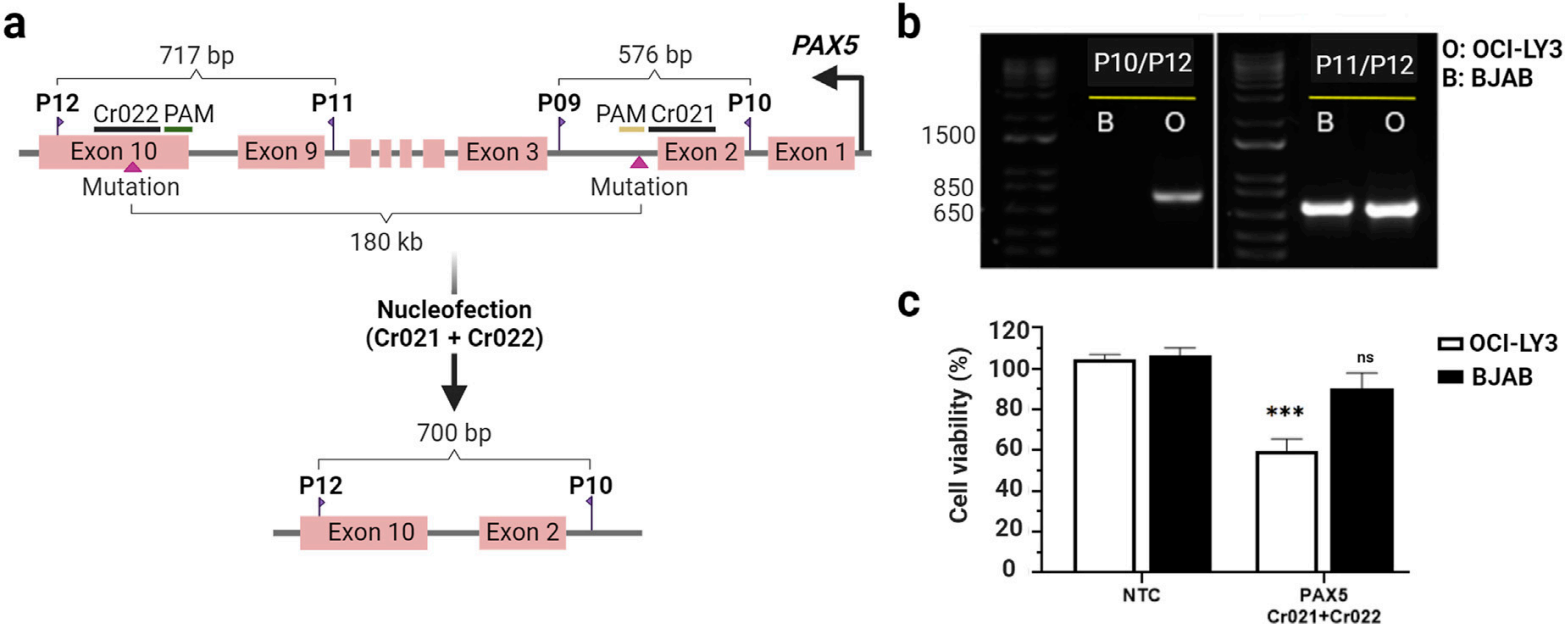
Knocking out of the *PAX5* gene using dual OCI-LY3 specific-crRNAsreduces cell viability. Schematic representation of *PAX5* exons, primer positions, and the locations of Cr021 (in exon two-intron junction) and Cr022 (in the last exon) **(A)**. OCI-LY3 and BJAB lymphoma cells were electroporated with Cr021/Cr022. Following a 72-h treatment, cells were collected, genomic DNA was isolated, and the target sequence regions were amplified with primers flanking the expected edited points. Agarose gel electrophoresis was performed to visualize the edited samples. Gel electrophoresis revealed PCR bands only in double-crRNAs edited OCI-LY3 cells (left gel), with no detectable PCR bands in BJAB cells, indicating no editing in these cells. Agarose gel electrophoresis using primers flanking Cr022 showed two PCR bands in OCI-LY3 (O) and BJAB **(B)** samples, confirming the presence of genomic DNA in both samples **(B)**. Cell viability was assessed as a percentage relative to the negative control in OCI-LY3 (white columns) and BJAB (black columns) cells electroporated with Cr021/Cr022. Results are presented as mean ± SEM; ***p < 0.001 **(C)**. Primer details: primer A (P10), primer B (P9), primer C (P12), and primer D (P11). This Figure was created by using biorender.com.

Editing efficiency was examined 72 h post-nucleofection via PCR using a combination of primers spanning the target sites of Cr021 and Cr022 (Figure 3A). The editing process is followed by recombination, which brings both edited genomic regions into proximity. Consequently, the presence of a PCR band indicates successful editing by both crRNAs, while its absence indicates the failure of editing by at least one crRNA. Editing was confirmed through PCR amplification, resulting in the generation of large deletion alleles (_∼_180 kbp). The absence of PCR bands in BJAB cells indicated a lack of gene editing (Figure 3B). These findings were consistent with 40% reduced cell viability observed in OCI-LY3 while no significant impairing effect on cell viability was detected in BJAB cells (Figure 3C). By simultaneously targeting distinct mutation sites, we not only slightly improved the cellular impact but also expanded the versatility of the approach by inducing large-scale deletions, offering another manner to deplete target proteins and induce cancer-specific depletion of cell viability.

### CRISPR/Cas9-mediated dual gRNA targeting of *PAX5* and *MYC* mutations induces chromosomal rearrangements in OCI-LY3 cells

**FIGURE 4:**
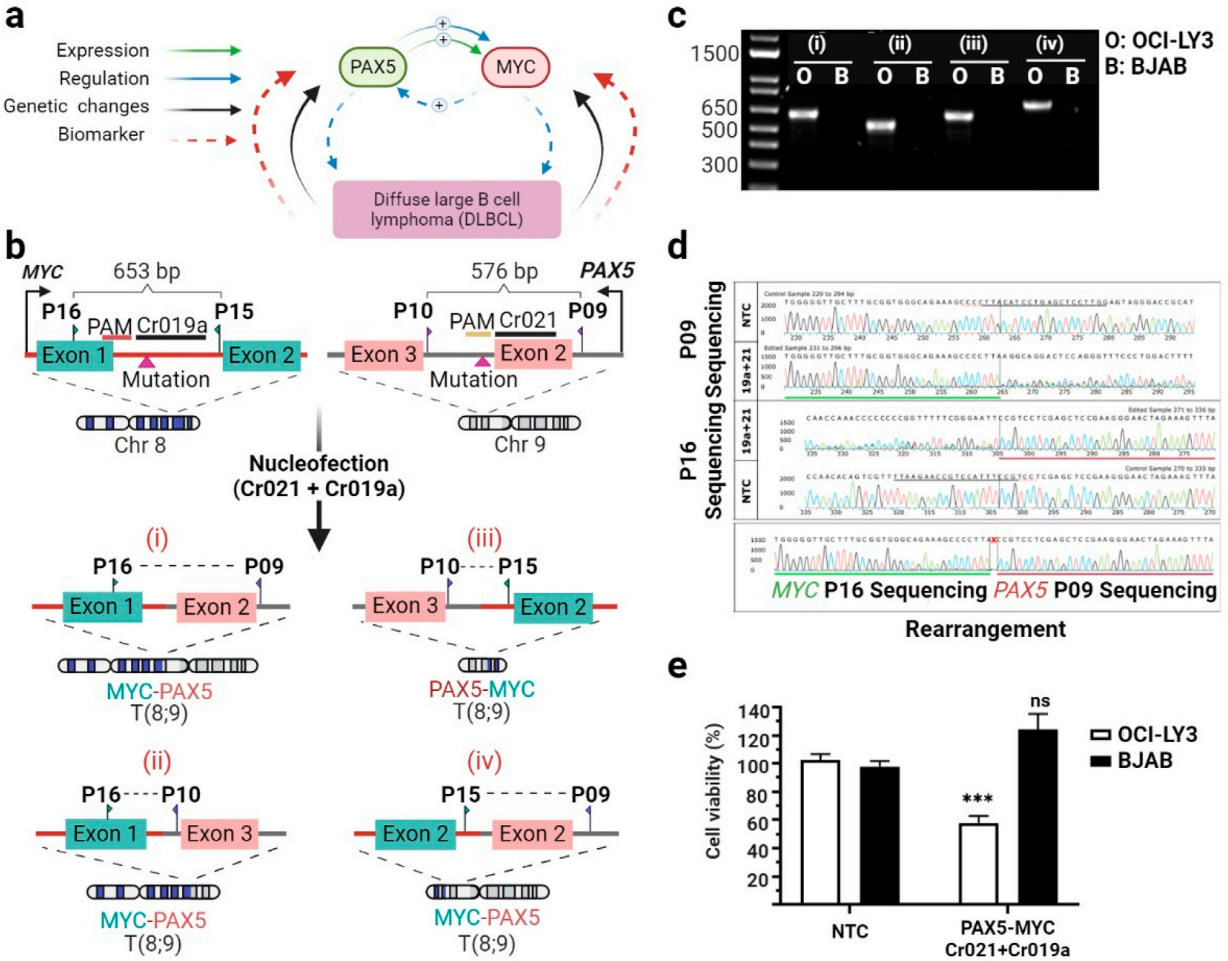
Using a dual-crRNA strategy to knock out the *PAX5* and *MYC* genes reduces cell viability. Molecular connections and clinical implications of *PAX5* and *MYC* in DLBCL **(A)**. Schematic representation illustrating the maps of the *PAX5* and *MYC* exons, primer positions, and the locations of Cr021 (targeting the exon two intron junction) and Cr019a (targeting intron 1). Four possible rearrangements upon combination of both crRNAs are depicted **(B)**. OCI-LY3 and BJAB lymphoma cells (2.5 × 10^5^ cells) were subjected to electroporation with Cr021 and Cr019a. After a 72-h treatment, cells were harvested, and genomic DNA was isolated. The target sequence regions were then amplified with primers flanking the expected edited points, followed by agarose gel electrophoresis to visualize the edited samples. The agarose gel electrophoresis depicts PCR-amplified products using forward and reverse external primers for four different chromosomal rearrangement possibilities. The PCR band is amplified only in double-crRNA edited OCI-LY3 (O) cells, while no amplification is observed in BJAB cells, indicating no editing in the latter **(C)**. PCR amplicons were subsequently subjected to Sanger sequencing. The traces depict the edited sequences compared to control cells treated with non-targeting crRNA (NTC). The *PAX5-MYC* rearrangement traces confirm the deletion of approximately 90 Mb of sequence, with the horizontal green underlined region representing *MYC* and the horizontal red underlined region representing *PAX5* **(D)**. Cell viability was determined as a percentage relative to the negative control in OCI-LY3 (white columns) and BJAB (black columns) cells electroporated with Cr021/Cr019a and Cr021/Cr022. Results are presented as mean ± SEM; ***p < 0.001 **(C)**. Primer details: Primer A (P15), Primer B (P16), Primer C (P10), and Primer D (P9) **(E)**. This Figure was created by using biorender.com.

Considering the potential of multiplexing the CRISPR/Cas system to delete large DNA regions in a mutations-specific fashion, we next explored the possibility of inducing chromosomal rearrangements between genes for functional inactivation. The strategy was based on the understanding of the intricate crosstalk between various cellular pathways and associated proteins in cancer cells. We concentrated on two genes, *PAX5* and *MYC*. *PAX5*, as demonstrated earlier, plays a crucial role in lymphoma pathogenesis. Meanwhile, *MYC*, a transcription factor and oncogene, is implicated in various cancers, including lymphomas, and is associated with aggressive clinical behavior. PAX5 has been shown to directly regulate MYC expression by binding to its regulatory regions, thereby modulating MYC-driven cellular processes (Medvedovic et al., 2011). Additionally, both PAX5 and MYC can be regulated by common signaling pathways, such as the B-cell receptor (BCR) and NF-κB pathways, further intertwining their expression and function in DLBCL (Liu et al., 2017) (Figure 4A). Hence, we combined two crRNAs, Cr021 targeting the *PAX5*, and Cr019a addressing *MYC*. Concretely, Cr019a was directed against the variant C>T at position 127,736,999 located in the first intron on chromosome 8. This variant was found to be homozygous in OCI-LY3 cells and heterozygous in the BJAB cell line.

Once again, gene editing was investigated using primers spanning the target sites of Cr019a and Cr021. These target loci are separated by a genomic sequence of _∼_90.7 Mb. PCR analysis demonstrated a large deletion and the induction of *MYC-PAX5* rearrangement between dual-edited OCI-LY3 cells. According to our expectations, no PCR product was obtained in control BJAB cells, indicating the absence of any *MYC-PAX5* rearrangement (Figures 4B, C). Moreover, dual gRNA treatment caused a 42% reduction of cell viability in target OCI-LY3 cells compared to untreated cells. In contrast, combination gRNA treatment did not impair the cell viability of control BJAB cells, consistent with our gene editing observations (Figure 4E).

This approach enables the perturbation of multiple driver genes located on different chromosomes, specifically targeting cancer-specific mutations without affecting cells lacking these mutations. Such manipulation of distinct cancer survival pathway networks may significantly enhance the susceptibility of cancer cells to therapeutic interventions.

### Cellular cause of reduced cell viability

Cell viability is dependent on both cell proliferation and cell death. The reported findings show a significant reduction in cell viability in OCI-LY3. This is the case when targeting *PAX5* with a single mutation-specific crRNA (Cr021), targeting *PAX5* with dual OCI-LY3 mutation-specific cRNAs (Cr021+Cr022), and when targeting *PAX5* and *MYC* with a dual-crRNA mutation-specific strategy (Cr021+Cr019a). Following this, we wanted to elucidate the cellular roots of the observed effect by measuring cell death and cell proliferation with cellular assays.

**FIGURE 5:**
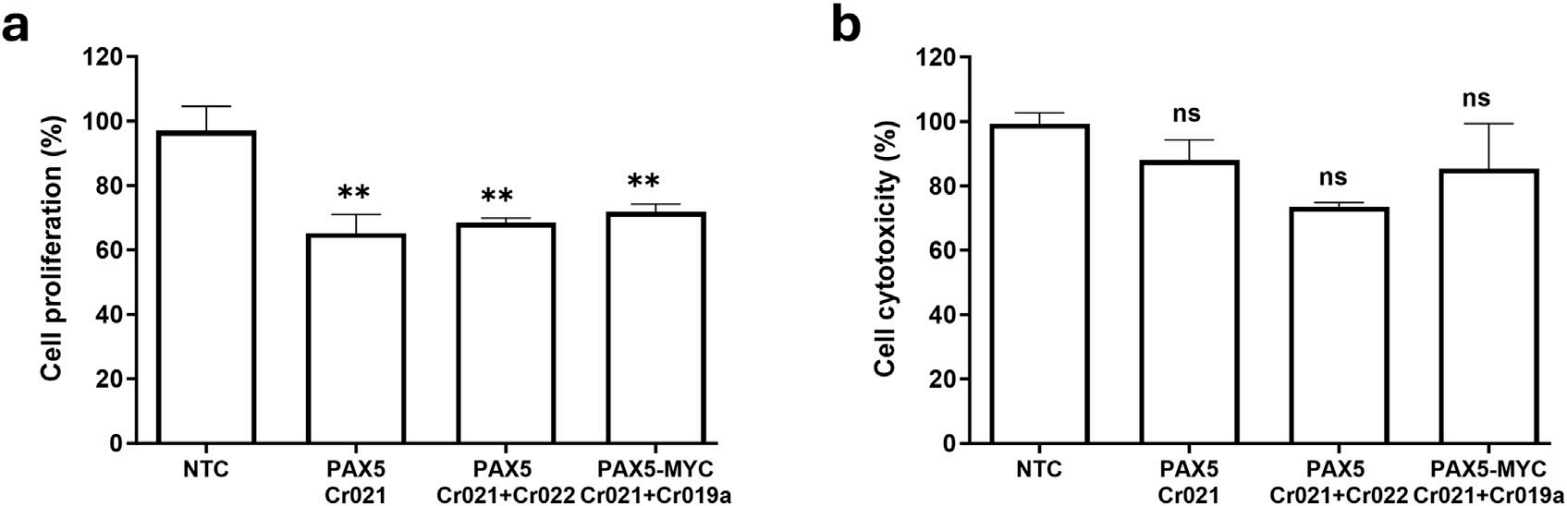
Results of cellular proliferation and cytotoxicity assays following gene knockout with a variety of CRISPR constructs. 2 × 10^5^ OCI-LY3 cells were subjected to electroporation with Cr021, Cr021+Cr022 and Cr021+Cr019a to target the genes *PAX5 or PAX5 + MYC*. Cell proliferation **(A)** and cell cytotoxicity **(B)** were determined after 72 h as a percentage relative to the non-targeting control (NTC) in OCI-LY3. Results are presented as mean of three experimental rounds ±SEM; n.s not significant, *p < 0.05, **p < 0.01.

Cell proliferation was stably reduced in all experimental rounds for all performed knockouts. The tested crRNAs elicited a reduction of proliferation between 27% and 35% compared to the non-targeting control (NTC). It can be concluded that cell proliferation is significantly reduced in all knockouts and is the driver of the observed cell viability reduction (Figure 5A).

The data for cell death, however, showed no statistically significant change for any of the performed knockouts (Figure 5B). Although non-significant, several rounds of nucleofection indicated rather a reduction in cell death compared to the reference sample. This result might coincide with reports of a dual oncogenic/tumor suppressive behavior of *PAX5* in the correct cell context. *PAX5* has been shown to act as a tumor suppressor in the B-lymphoid lineage (Cobaleda et al., 2007) and haploinsufficiently synergize with Stat5b-CA to induce ALL in mice (Heltemes-Harris et al., 2011). It is feasible that a knockout might act anti-apoptotically in some cases, yet more research is required for a conclusive statement.

These results lead us to conclude that the observed reduced cell viability (Figure 2C; Figure 3C; Figure 4E) following a knockout of *PAX5* or combination of *PAX5* and *MYC* can be clearly attributed to a reduction in cell proliferation, while cell death remains largely unaffected.

### Functional consequences of single targeting introns in DLBCL cells

**FIGURE 6:**
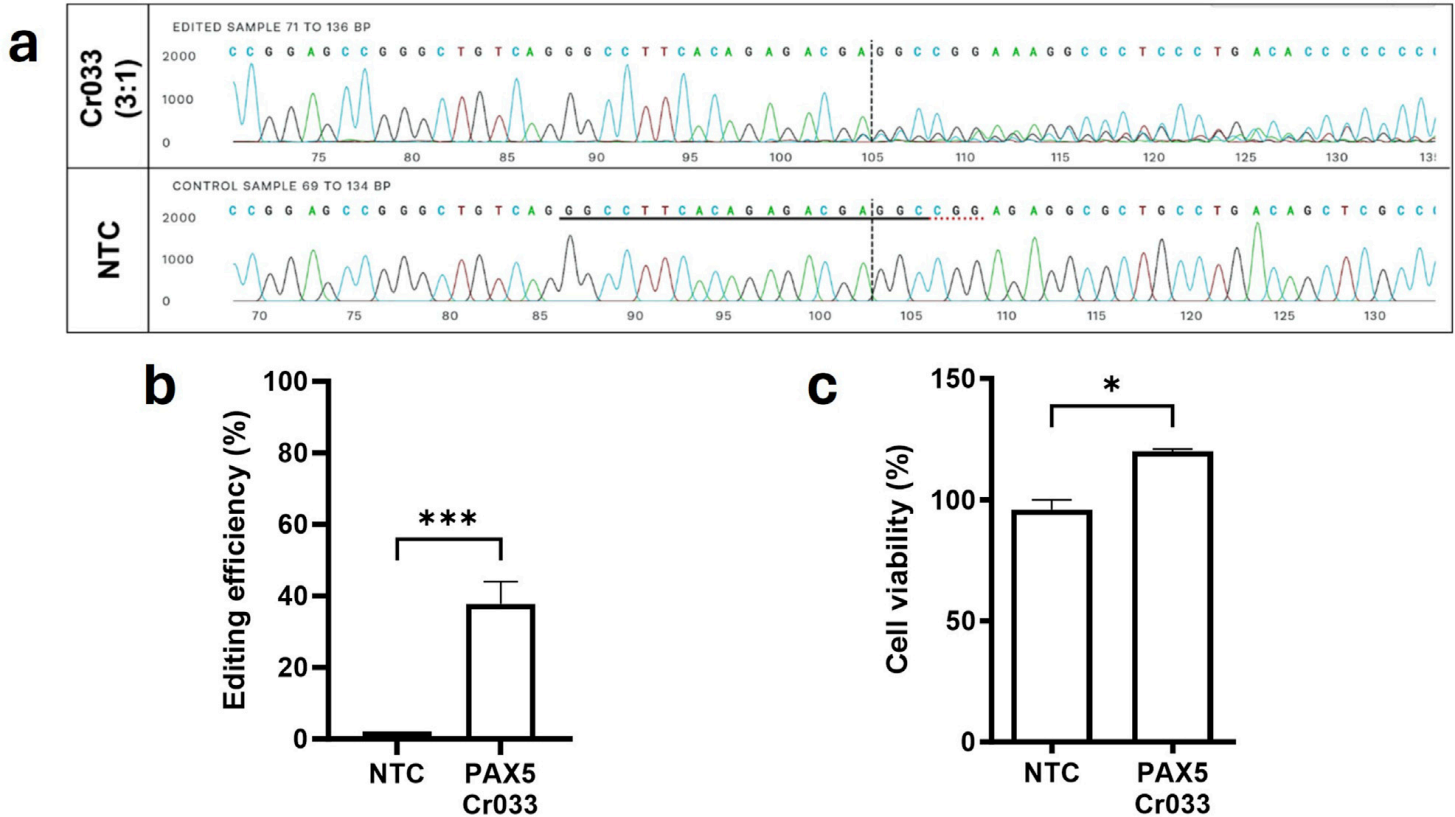
Targeting mutations in late exons and intronic genomic regions did not result in loss of function or cancer cell death. Cr033 was designed to target a mutation within an intron of the *PAX5* gene specific to OCI-LY3 cells. We electroporated 2.5 × 10^5^ lymphoma cells with 5 µM of each crRNA. After 72 h of treatment, cells were collected, genomic DNA was isolated, and target sequence regions were amplified and sequenced. Gene editing efficiency was determined by comparing Sanger sequences with wild-type (control) cells. The horizontal black underlined region represents the guide sequence, while the horizontal dotted underline indicates the PAM site. The cut site is indicated by a vertical black dotted line **(A)**. Total editing efficiencies were calculated by comparing the sequencing chromatogram of the test sample with control cells treated with non-targeting crRNA (NTC) using the ICE web tool **(B)**. Cell viability of target cells compared to control cells treated with NTC was assessed **(C)**. Results are shown as mean ± SEM, (*p < 0.05, ***p < 0.001).

Lastly, we aimed to investigate the effect of solely targeting intronic gene regions of driver genes on the viability of OCI-LY3 lymphoma cells. We utilized Cr033, which targets the *PAX5* variant A>G at position 36,864,872 located within the second intron of chromosome 9. This variant was found to be homozygous in OCI-LY3 cells. Despite achieving almost 40% gene editing efficiency (Figures 6A, B), targeting this intronic mutation did not lead to a reduction in cell viability (Figure 6C). This observation suggests that inducing single double-strand breaks within introns may not necessarily result in a depletion of viable cancer cells, underscoring the importance of considering the functional consequences of genetic alterations in cancer cells.

Multiple other mutations were investigated in varying intronic regions of both cell lines in similar experiments. Yet, no reduction of viability could be detected, evidencing that simply targeting arbitrary mutated regions within a gene may not suffice to deplete cancer cell viability (data not shown). Instead, we hypothesize that it may be critical to disrupt the function of the encoded protein to effectively impede tumor growth and survival. Overall, these experiments highlight the complexity of cancer biology and emphasize the necessity for targeted approaches that specifically disrupt oncogenic pathways to achieve therapeutic efficacy.

## Discussion

Our study demonstrates the efficacy of programmable nucleases, specifically CRISPR/Cas9, in targeting specific cancer-causing genomic sequences *in vitro*. While previous studies have shown the disruption of various cancer-associated mutations, our focus on targeting oncogenes such as *PAX5*, *MYC*, and *CD79B* in diffuse large B-cell lymphoma (DLBCL) represents a significant advancement (Honeywell et al., 2023; Montalbano et al., 2017). This approach enables precise manipulation of essential genes implicated in cancer pathogenesis, offering a potential strategy to combat molecular heterogeneity in cancer. It presents a promising alternative to traditional therapies, which frequently target downstream changes or rely on less specific mechanisms of action. Importantly, by selectively targeting cancer cells while preserving normal cells, CRISPR/Cas minimizes off-target effects, thereby lowering the risk of adverse reactions.

By contrast with other approaches where cancer cell death or reduced cancer cell viability is reached by simultaneously employing up to 50 sgRNAs (Kwon et al., 2022b), our study shows the feasibility of selecting mutations systematically and methodically to deplete malignant cells in a specific and effective manner. A requirement therefore may be to disrupt domains relevant to protein function. Upcoming studies will focus on deepening and clarifying the relationship between the silencing of certain protein domains and the triggering of cancer cell death. While we provide evidence that mutation-specific gRNAs successfully reduce cell viability, these are not frequently occurring mutations in DLBCL patients. Yet, the oncogenes investigated in this study are known to be frequently mutated in these patients, increasing the clinical significance of the strategy. We substantiate the impact of the target location, favoring early mutations interrupting the protein function, as well as the necessity of targeting essential oncogenes, as the mere disruption of non-essential genes does not suffice to impair cancer cell proliferation. Additionally, we provide insights into addressing the challenge of identifying actionable mutations in cancer patients (Alaggio et al., 2022). We demonstrated that, for instance, if mutations are not found in early exons of the driver genes for a patient, a dual-system approach can be employed. This involves using two different gRNAs specific to a given intron or exon to disrupt important protein domains, thereby expanding the scope of targetable genomic alterations. This approach not only enhances the precision of CRISPR/Cas targeting but also enables the identification of mutations located in regions that may have been previously overlooked. By broadening the range of targetable mutations, we increase the likelihood of achieving therapeutic efficacy in patients with diverse mutational profiles, ultimately advancing the goal of personalized cancer treatment. By targeting multiple mutations, we aim also to disrupt various oncogenic pathways simultaneously, potentially achieving more comprehensive and durable treatment responses. It was shown that the reduction of cell viability could be traced back to an inhibition of cell proliferation. While this is the case for the genes knocked out in this study, it is plausible that knockout of other central genes in DLBCL results in cancer cell death or a mixture of both, dependent on the unique pathways affected. Furthermore, the transient delivery of Cas9 and gRNA allows for permanent target gene disruption, offering the potential for long-lasting therapeutic effects. This feature distinguishes CRISPR-Cas from traditional RNA-based therapies that require continuous administration to maintain efficacy.

While our study represents a proof-of-concept for targeting driver genes in DLBCL, there are also challenges such as incomplete editing efficiency and delivery limitations (Katti et al., 2022). These factors emphasize the need for further optimization of CRISPR/Cas delivery methods and the development of strategies to enhance editing efficiency *in vivo*. Engineered lipid nanoparticles (LNPs) and virus-like particles represent promising strategies for the effective delivery of therapeutic proteins *in vivo* (Rosenblum et al., 2020; Banskota et al., 2022). Further endeavors will enhance the targeted delivery of the proteins in malignant cells, which will improve the therapeutic efficacy while diminishing side effects. Additionally, the potential for immune responses to Cas9 proteins warrants careful consideration in clinical applications (Roy, 2021). DLBCL patients unresponsive to standard treatment regimens and patients exhibiting drug resistance or disease remission could benefit particularly from such a personalized strategy. Moreover, our study acknowledges the existence of additional driver genes in DLBCL, such as *ACTB*, *BTG2*, *PLET1*, *CARD11*, and *DIXDC1*, which could be potential targets for future investigation (Fan et al., 2020). Addressing these targets may further enhance the efficacy and precision of CRISPR-based therapies in DLBCL. Another aspect to be considered in future studies is the divergence between both DLBCL forms, the germinal center and the activated B-cell-like subtypes, characterized by diverse gene expression profiles and enrichment of distinct mutational events. Upcoming investigations may shed light on the transferability of the present approach to a wider range of cell lines and patient samples.

In summary, our findings open avenues for further exploration of CRISPR/Cas in cancer therapy, offering insights into both treatment strategies and the underlying biology of oncogenic drivers. By addressing molecular heterogeneity and leveraging the specificity and recent versatility of CRISPR/Cas, we move closer to realizing the promise of precision medicine in cancer treatment.

## Data Availability

The original contributions presented in the study are included in the article/Supplementary Material, further inquiries can be directed to the corresponding author.

## Supplementary material

SUPPLEMENTARY FIGURE S1 Mutation position in guide RNAs and Sanger sequencing validation. For each CRISPR guide RNA (Cr021, Cr022, Cr033, Cr019a), the Sanger sequencing results were analyzed in both cell lines, BJAB and OCI-LY3. In the sequencing chromatograms, the blue triangle indicates the position of the mutation, while the red arrows indicate the position of the gRNA.

SUPPLEMENTARY FIGURE S2 Optimization of crRNA concentration and time point for CRISPR/Cas9 editing in OCI-LY3 cells. Following electroporation of OCI-LY3 cells with Cr012gRNA:Cas9 ratio of 1:1 (gRNA conc. 1.65 µM), 2:1 (gRNA conc. 3.4 µM), and 3:1 (gRNA conc. 5 µM), genomic DNA was isolated after 24 h, 48 h, and 72 h. Amplification of target sequence regions was performed using primers flanking the expected edited points, followed by Sanger sequencing. Editing efficiencies were calculated by comparing sequencing chromatograms of test samples with control cells treated with non-targeting crRNA (NTC) using the ICE web tool.
