# Supplementary figures and images for: Targeting DLBCL by mutation-specific disruption of cancer-driving oncogenes
Source: Front Genome Ed. 2024 Oct 14;6:1427322. doi: 10.3389/fgeed.2024.1427322 (PMC11513324; doi:10.3389/fgeed.2024.1427322)

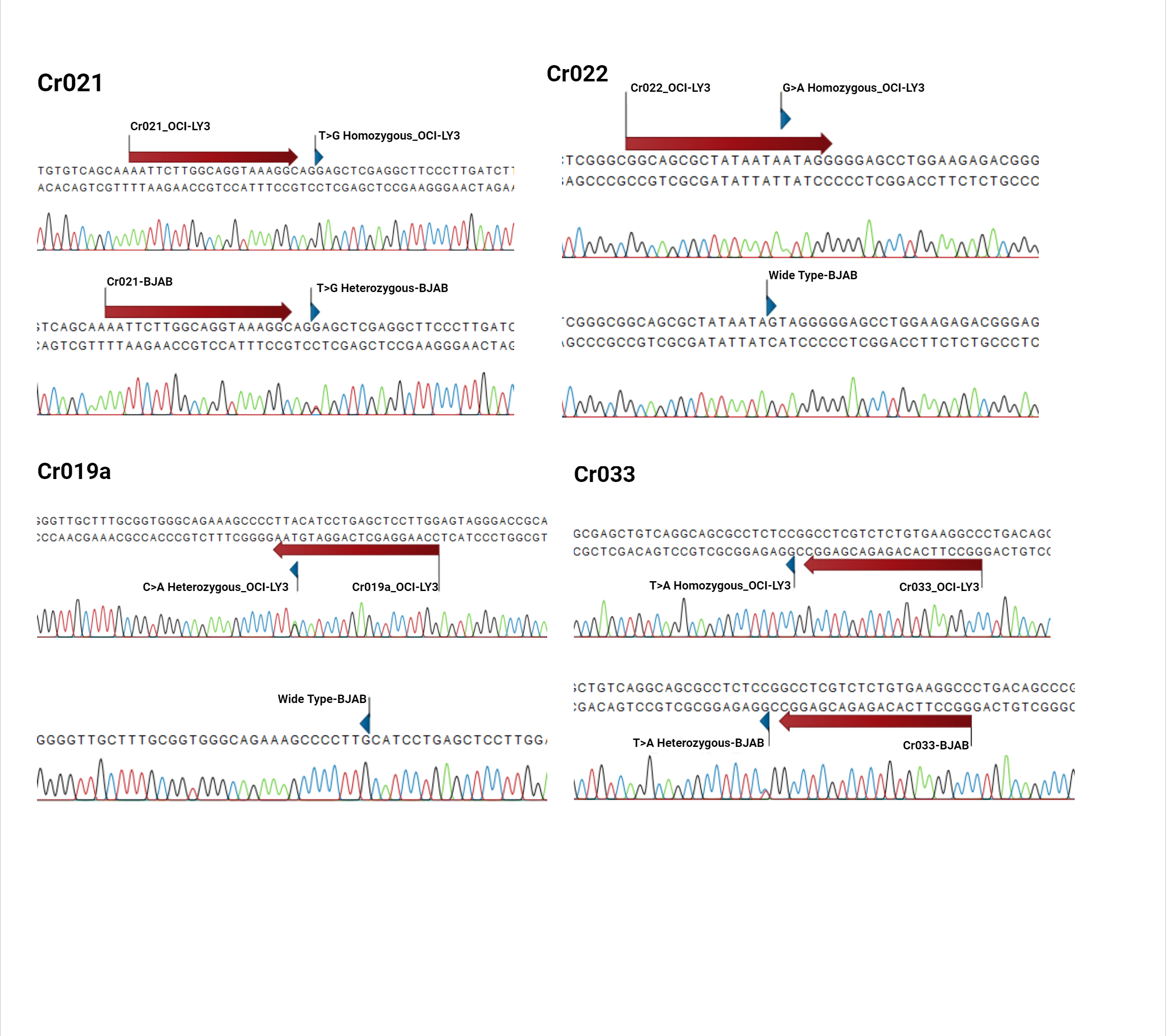

Supplement: Supplementary file 3 [file Image1.JPEG]

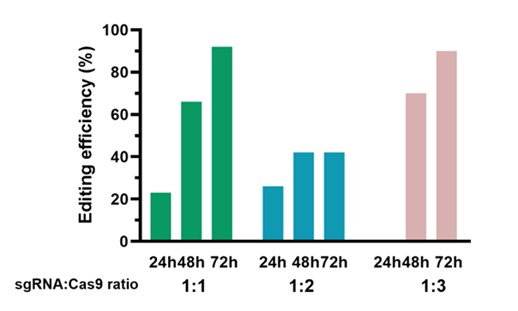

Supplement: Supplementary file 4 [file Image2.JPEG]
